# Heavy water coupling gel for short-wave infrared photoacoustic imaging

**DOI:** 10.1117/1.JBO.28.11.116001

**Published:** 2023-11-14

**Authors:** Christopher M. Salinas, Eric Reichel, Abhiman Gupta, Russell S. Witte

**Affiliations:** a University of Arizona, College of Optical Sciences, Tucson, Arizona, United States; b University of Arizona, Department of Biomedical Engineering, Tucson, Arizona, United States; c University of Arizona, Department of Medical Imaging, Tucson, Arizona, United States

**Keywords:** high resolution ultrasound, photoacoustic imaging and spectroscopy, optoacoustic imaging, short-wave infrared, heavy water, lipids, collagen, cancer

## Abstract

**Significance:**

Changes in lipid, water, and collagen (LWC) content in tissue are associated with numerous medical abnormalities (cancer, atherosclerosis, and Alzheimer’s disease). Standard imaging modalities are limited in resolution, specificity, and/or penetration for quantifying these changes. Short-wave infrared (SWIR) photoacoustic imaging (PAI) has the potential to overcome these challenges by exploiting the unique optical absorption properties of LWC>1000  nm.

**Aim:**

This study’s aim is to harness SWIR PAI for mapping LWC changes in tissue. The focus lies in devising a reflection-mode PAI technique that surmounts current limitations related to SWIR light delivery.

**Approach:**

To enhance light delivery for reflection-mode SWIR PAI, we designed a deuterium oxide ($D_{2}O$, “heavy water”) gelatin (HWG) interface for opto-acoustic coupling, intended to significantly improve light transmission above 1200 nm.

**Results:**

HWG permits light delivery >1  mJ up to 1850 nm, which was not possible with water-based coupling (>1  mJ light delivery up to 1350 nm). PAI using the HWG interface and the Visualsonics Vevo LAZR-X reveals a signal increase up to 24 dB at 1720 nm in lipid-rich regions.

**Conclusions:**

By overcoming barriers related to light penetration, the HWG coupling interface enables accurate quantification/monitoring of biomarkers like LWC using reflection-mode PAI. This technological stride offers potential for tracking changes in chronic diseases (*in vivo*) and evaluating their responses to therapeutic interventions.

## 1 Introduction

Changes in tissue content of lipids, water, and collagen (LWC) play a role in the development of numerous diseases and their response to therapy. For example, deficiencies in lipid transport and structure in the brain are linked to early-onset Alzheimer’s disease.^1^^,^^2^ Also, changes in lipid content and collagen are associated with arterial blockages and plaque buildup in atherosclerosis.^3^ Finally, compositional changes of lipids and water in the tumor microenvironment are known to occur during development and progression of certain types of cancer.^4^ It is, therefore, highly desirable to develop noninvasive technology capable of detecting and monitoring these compositional changes. Standard imaging modalities, including magnetic resonance imaging (MRI), computed tomography (CT), ultrasound (US) and most optical methods are limited in their ability to quantify these changes at high resolution deep into tissue;^5^ MRI and CT lack the sensitivity and specificity to quantify LWC with sub-mm precision. High-resolution optical techniques like fluorescence (two photon and confocal) and optical CT are limited in their depth of penetration by the optical diffusion limit (∼1  mm). While pulse echo (PE) ultrasound benefits from sub-mm spatial resolution, it contains poor contrast for discerning different types of soft tissue structures and molecules since the images rely on small differences in acoustic impedance. Therefore, none of these modalities are ideal in terms of resolution, penetration, specificity, field of view, and acquisition time.

**Fig. 1:**
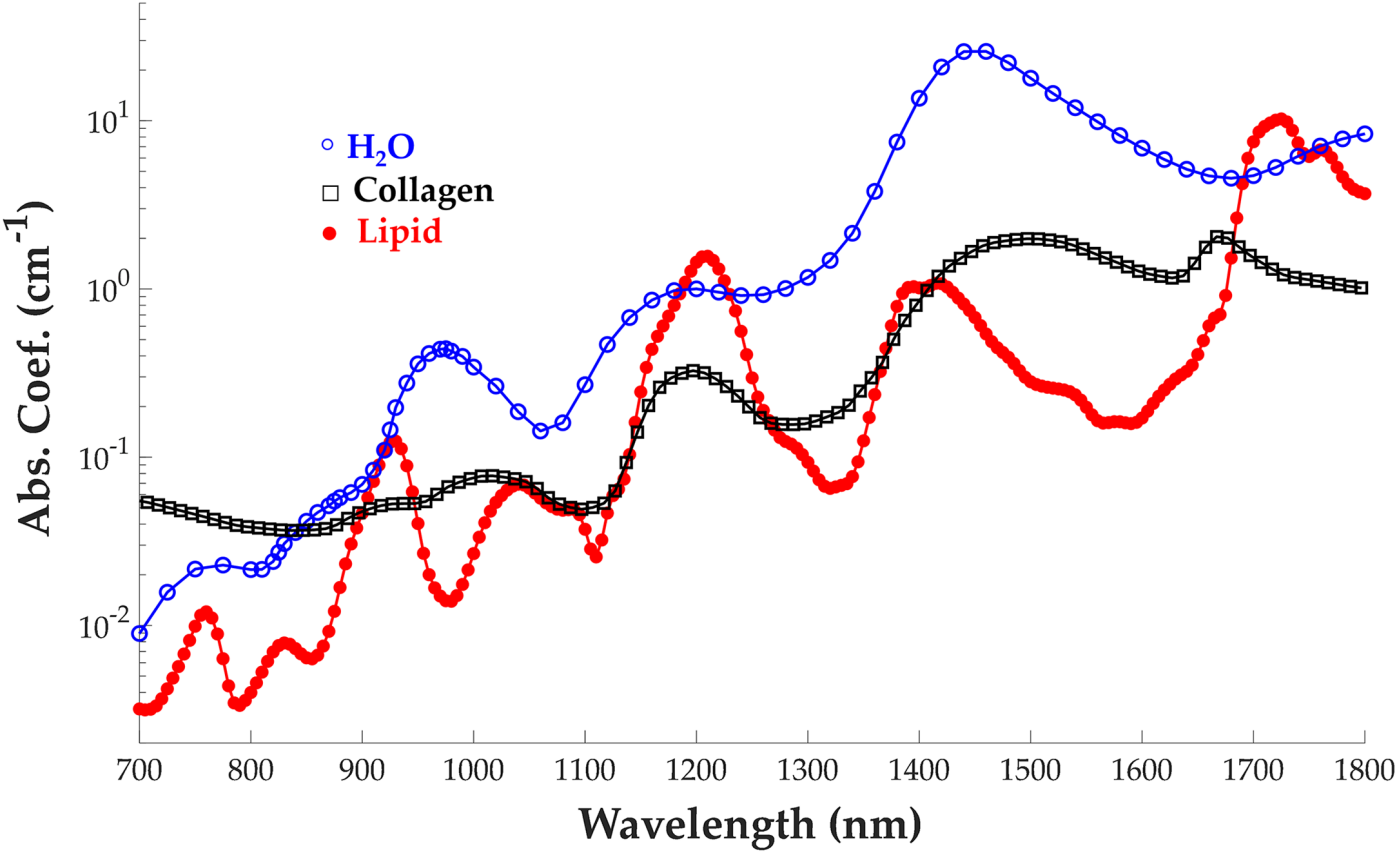
Optical absorption coefficients for lipid, water, and collagen between 700 and 1800 nm.^18^^–^^20^

Photoacoustic imaging (PAI), on the other hand, benefits from high resolution, high contrast, and real-time acquisition compared to traditional biomedical imaging modalities. PAI depends on a local and transient heating effect offering greater penetration (at least 10 mm) than other optical methods that depend on coherent illumination. Traditional PAI uses visible and near infrared (VIS/NIR, 400 to 1000 nm) light to detect endogenous chromophores like hemoglobin and absorbing dyes and nanoparticles like indocyanine green and gold nanorods.^6^^–^^8^ Other optical methods have been employed in this waveband to quantify LWC content in tissue,^9^^–^^11^ however, at superficial tissue depths <1  mm. Additionally, these absorbers are notoriously challenging to quantify in the VIS/NIR window due to their relatively weak optical absorption.^12^ Several commercially available PAI systems^13^^,^^14^ are only sensitive to chromophores whose absorption exceeds $∼0.1\;{\mathrm{cm}}^{-1}$. This leads to images with poor signal-to-noise ratio (SNR), limited depth penetration and/or requires light that exceeds the safety limit.^15^^–^^17^ Conversely, PAI in the short-wave infrared (SWIR, 1200 to 2000 nm) holds promise for quantifying and mapping endogenous absorbers like LWC due to their much higher absorption (Fig. 1) and unique spectral features in this band.

**Fig. 2:**
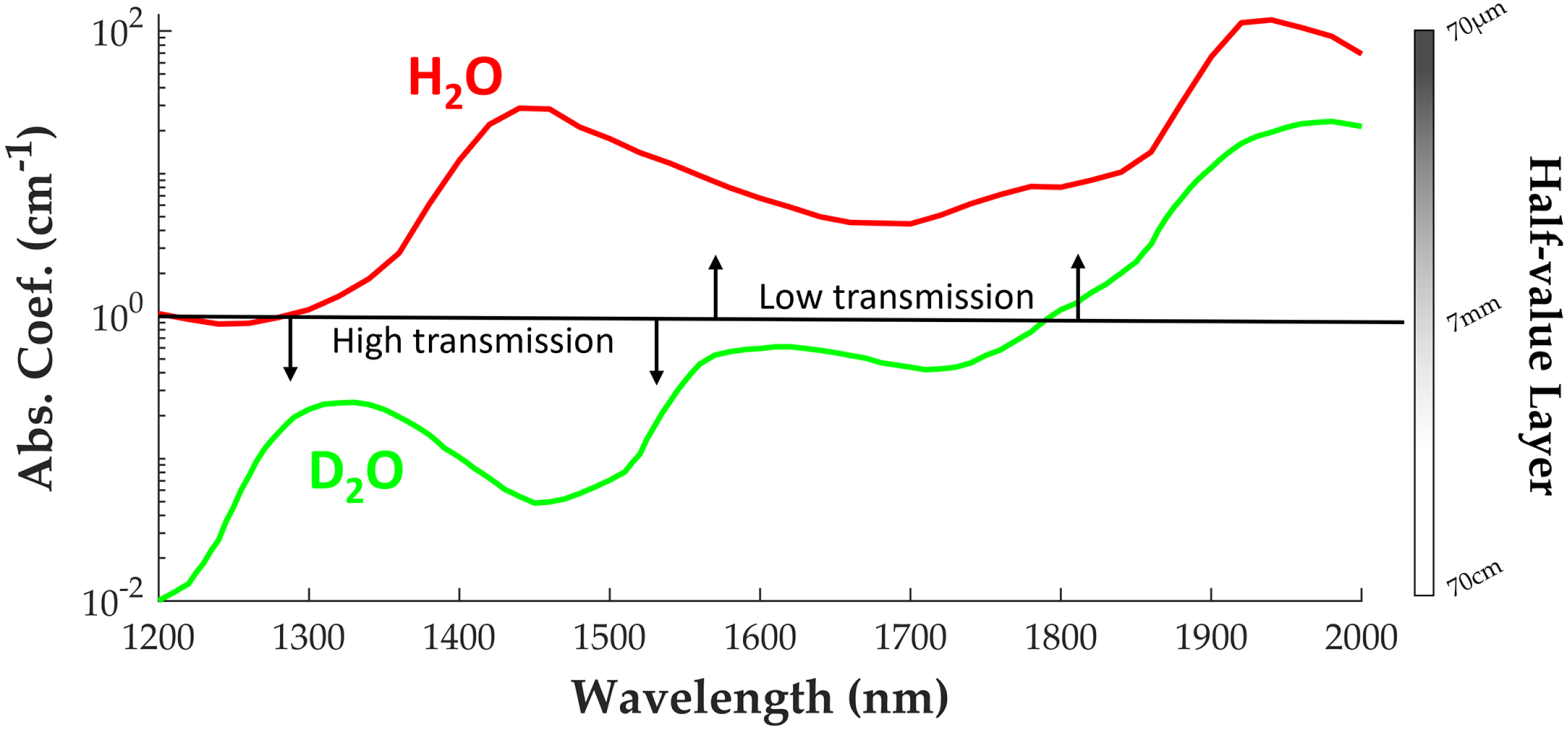
SWIR absorption coefficients for water ($H_{2}O$) and heavy water ($D_{2}O$) between 1200 and 2000 nm. Half-value propagation distance dramatically decreases above 1200 nm for $H_{2}O$ compared to $D_{2}O$.^20^

Techniques that employ PAI in the SWIR have previously demonstrated capability to probe lipid and collagen changes in diseases such as atherosclerosis and cancer.^21^^,^^22^ While lipid and collagen profiles in these diseases are well documented, rigorous quantification of lipid changes during Alzheimer’s progression is an ongoing endeavor^23^^,^^24^ that SWIR PAI could address.^25^^,^^26^ However, the strong optical absorption of traditional optical and acoustic coupling media (e.g., water, oils, and rubbers) above 1200 nm have posed a significant challenge for reflection-mode (i.e., light and sound delivery from the same direction) PAI in the SWIR, which is desirable for *in vivo* imaging.^27^^–^^30^ Probing the optical properties of biological tissues is a necessary requirement for studies pertaining to LWC and other absorbers in the SWIR.^31^ In addition to poor optical transmission, strong dispersion and ultrasound attenuation of oil-based rubbers (e.g., humimic and polydimethylsiloxane) degrades PA and PE image quality, especially at high frequencies >20  MHz.^32^ The lack of an efficient coupling medium for reflection-mode PAI in the SWIR, therefore, poses a significant challenge for *in vivo* studies designed to detect and monitor endogenous contrast like LWC and other biomarkers of chronic diseases, such as cancer, atherosclerosis, and Alzheimer’s. A coupling agent that exhibits favorable optical and acoustic transmission properties would aid and improve such studies by increasing the overall signal throughput across a wide range of wavelengths, leading to more accurate characterization of constituents, such as LWC with strong absorption in the SWIR. Deuterium oxide ($D_{2}O$), commonly known as “heavy water,” is an isotope of $H_{2}O$ and exhibits unique and favorable properties for enabling reflection-mode PAI in the SWIR. Fig. 2 highlights the optical clarity of heavy water when compared to normal water, which is used as a base for many coupling materials. While several studies have demonstrated advantages of $D_{2}O$ coupling for PAI,^30^^,^^33^^–^^36^ there is a lack of a thoroughly well-characterized $D_{2}O$-based gelatin to facilitate reflection-mode PAI in the SWIR. This study addresses this limitation by developing and assessing the performance of a gel form of $D_{2}O$ for optimal delivery of light and ultrasound to enable reflection-mode PAI in the SWIR with a penetration of several millimeters into tissue. Compared to liquid, a gelatin interface simplifies coupling to the sample for reflection-mode imaging and eliminates the potential of leakage or formation of air bubbles. A gelatin coupling medium can also be reused and reshaped to conform to different imaging configurations. In addition, the advantage of tunability of gelatin stiffness further provides potential for construction of impedance matching layers, which can drastically improve ultrasound propagation to and from an imaging sample.^37^ The acoustic impedance of heavy water is approximately 1.64 MRayl at standard temperature and pressure, which is 10% higher than that of normal water given its increased density ($1.11\;g/{\mathrm{cm}}^{3}$) yet similar speed of sound.^38^ With the average acoustic impedance of soft tissue of ∼1.54  MRayl,^39^ heavy water coupling should provide similar ultrasound transmission to that of water (1.48 MRayl).

Not solely limited to endogenous chromophores, the advancement of reflection-mode PA systems in the SWIR would also allow for detecting and monitoring exogenous contrast agents with strong and potentially tunable absorption peaks in the SWIR.^31^ In this paper, we report a gelatin-based heavy water opto-acoustic coupling medium and interface designed for reflection-mode PE and PA imaging in the SWIR. We predict that a heavy water gelatin (HWG) will have a similar transmission profile to its liquid counterpart, which would enable *in vivo* PA studies above 1200 nm that require optical and acoustic coupling to the animal or human subject.

## 2 Methods

### 2.1 Fabrication of Heavy Water Gellan

Commercially acquired $D_{2}O$ (United Nuclear, 99% purity) and gellan gum (Modernist Pantry, “F,” low-acyl) were used for preparing HWG. Gellan gum was chosen as the gelling agent, as opposed to standard agarose, due to its better optical transparency and efficient acoustic transmission properties as internally studied and reported in literature by the co-authors.^40^ Gellan gum powder (between 2% and 3% w/w) was poured slowly into $D_{2}O$ preheated to ∼80°C and mixed with a stir bar to ensure homogeneity. The solution was then degassed to remove air bubbles before it was poured into a molding apparatus designed for reflection-mode PA and PE imaging. The mixture was allowed to cool to room temperature and ready for imaging studies. Because the stiffness and viscosity of the resulting HWG could be altered by adjusting the concentration of gellan gum, we determined that a concentration of 2.25% w/w gellan gum was suitable for imaging experiments because it provided mechanical stability while remaining somewhat flexible when coupling to the samples. The thickness of the gelatin coupling samples was ∼5.0  mm for reflection-mode imaging.

### 2.2 Optical and Acoustic Characterization

**Fig. 3:**
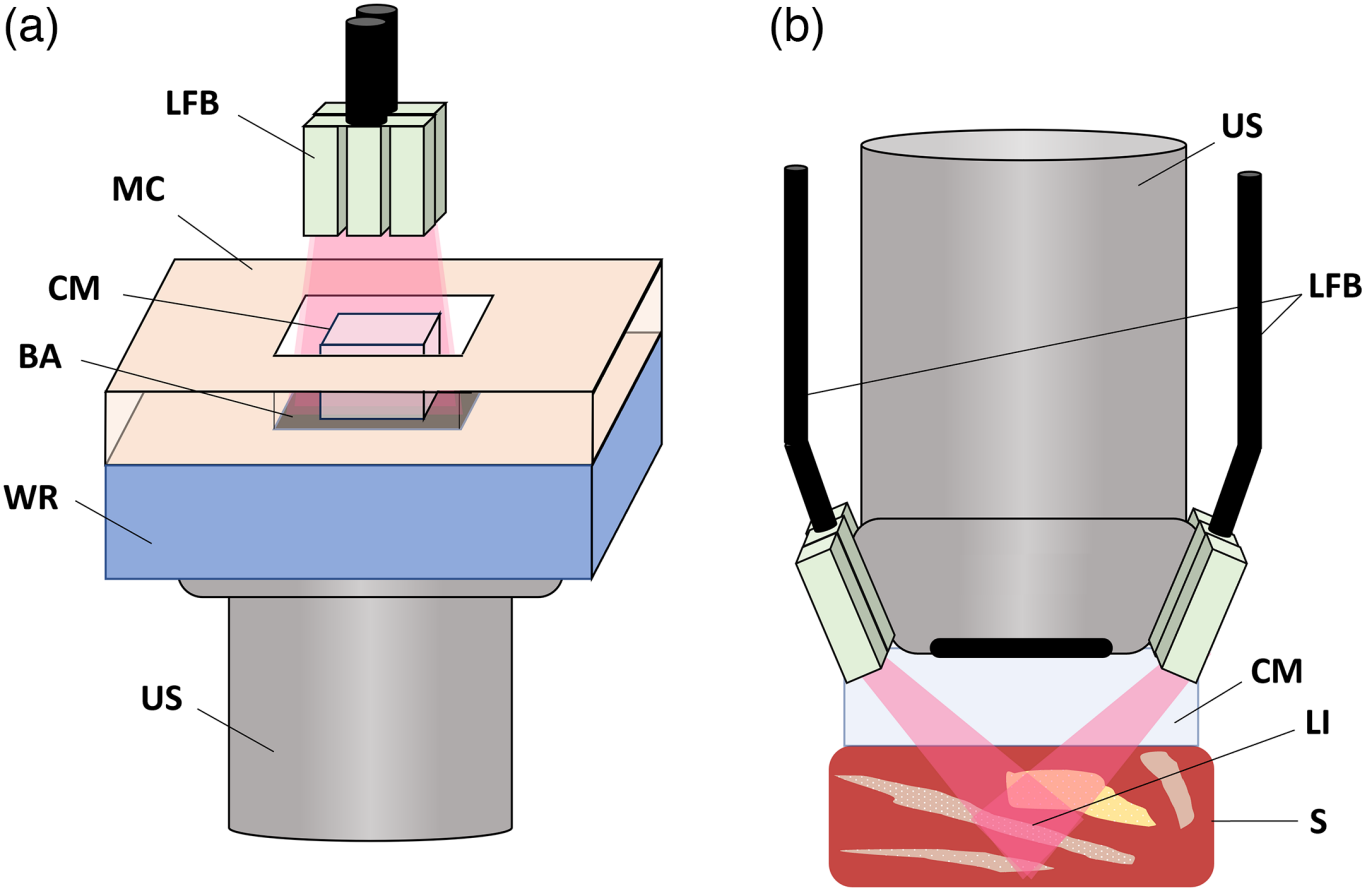
(a) Transmission-mode set up of the Vevo LAZR-X using the MX250 probe with LFB = laser fiber bundles, MC = 3D printed molding container, CM = coupling medium, BA = broadband absorber/electrical tape, WR = water reservoir, and US= linear array US probe. 2% and 3% w/w HWG samples are illuminated from above, and the resulting PA spectrum of the broadband absorber is used to quantify optical loss through the optical path. Acoustic coupling is achieved via the water reservoir in contact with the US array. (b) Standard reflection-mode setup with the coupling medium inserted between the probe and sample with LI = laser illumination pattern and S = sample.

A commercial ultrasound and PA imaging system (Vevo 3100/LAZR-X, VisualSonics) was first used in transmission-mode to quantify the optical loss through sections of HWG in the SWIR. Two small cubic samples (10×10×5  mm) of HWG were prepared (2% and 3% w/w) within a molding container displayed in Fig. 3(a). The container was placed within the output path of the fiber bundles, where acoustic coupling was then achieved from below via contact with a water reservoir and a 25 MHz US linear array (MX250, VisualSonics). Black electrical tape was used as a broadband optical absorber and inserted between the molding container and water reservoir as a baseline for estimating PA signal loss as a function of wavelength through the coupling medium. The spectrum of the tape was first measured through air in transmission-mode from 1200 to 2000 nm. The PA spectrum was then compared differentially to the broadband spectrum of the tape obtained through an optical path length defined by the thickness of HWG. Differences in these spectra result in the transmission loss corresponding to the new optical pathlength (i.e., HWG samples at 2% and 3% w/w). This method was repeated for samples of $H_{2}O$ gellan gum (WG) to compare the optical loss with HWG. The energy exiting the fiber bundle through the coupling media was also measured with a commercial energy meter (Coherent EnergyMax).

To determine whether HWG coupling affected acoustic propagation, we also calculated the point spread function (PSF) for standard PE imaging using the reflection-mode setup, as depicted in Fig. 3(b), using samples of HWG, WG and humimic medical rubber as coupling. Before the gel solidified, graphite powder (Loudwolf, 44  μm diameter) was distributed into the solution and allowed to cool to room temperature. These PSF images were used to estimate the axial resolution of the system through each coupling medium. Axial full-width half-maximum (FWHM) was chosen for measurement as it most closely depends on the US wavelength and dispersion (i.e., frequency-dependent attenuation of the US signal), unlike lateral and elevational resolution, which can depend on additional factors, including aperture size and focal distance. All PE and PA analyses were done on processed image data provided by the Vevo LAZR-X.

### 2.3 Sample Preparation

A lipid/water phantom composed of 20% lipid shortening (Cisco), 75% ${\mathrm{diH}}_{2}O$, and 5% w/w agarose was prepared to demonstrate the capabilities of HWG in reflection-mode PAI operating in the SWIR compared to a WG system. The solution of lipid, water, and agarose is brought to ∼80°C via hotplate, mixed with a stir bar, and left to cool and solidify at room temperature. HWG and WG coupling agents are molded for reflection-mode imaging with the 25 MHz probe and fiber bundle as described previously. Spectral PA data was collected at 5 nm intervals from 1200 to 2000 nm through both HWG and WG to quantify differences in SNR and detection. As a final validation of this reflection-mode PAI in the SWIR, a fresh sample of bovine muscle was used to demonstrate detection of intra-muscular lipid in the SWIR with HWG. Sections of locally sourced bovine tissue were cut into 10×10×10  mm cubes for full spectrum reflection-mode PA and PE imaging. The PA signal amplitude as a function of depth was estimated by integrating across the lipid/water phantom in the lateral (i.e., azimuth) direction for both HWG and WG datasets.

## 3 Results

### 3.1 Optical and Acoustic Characterization

**Fig. 4:**
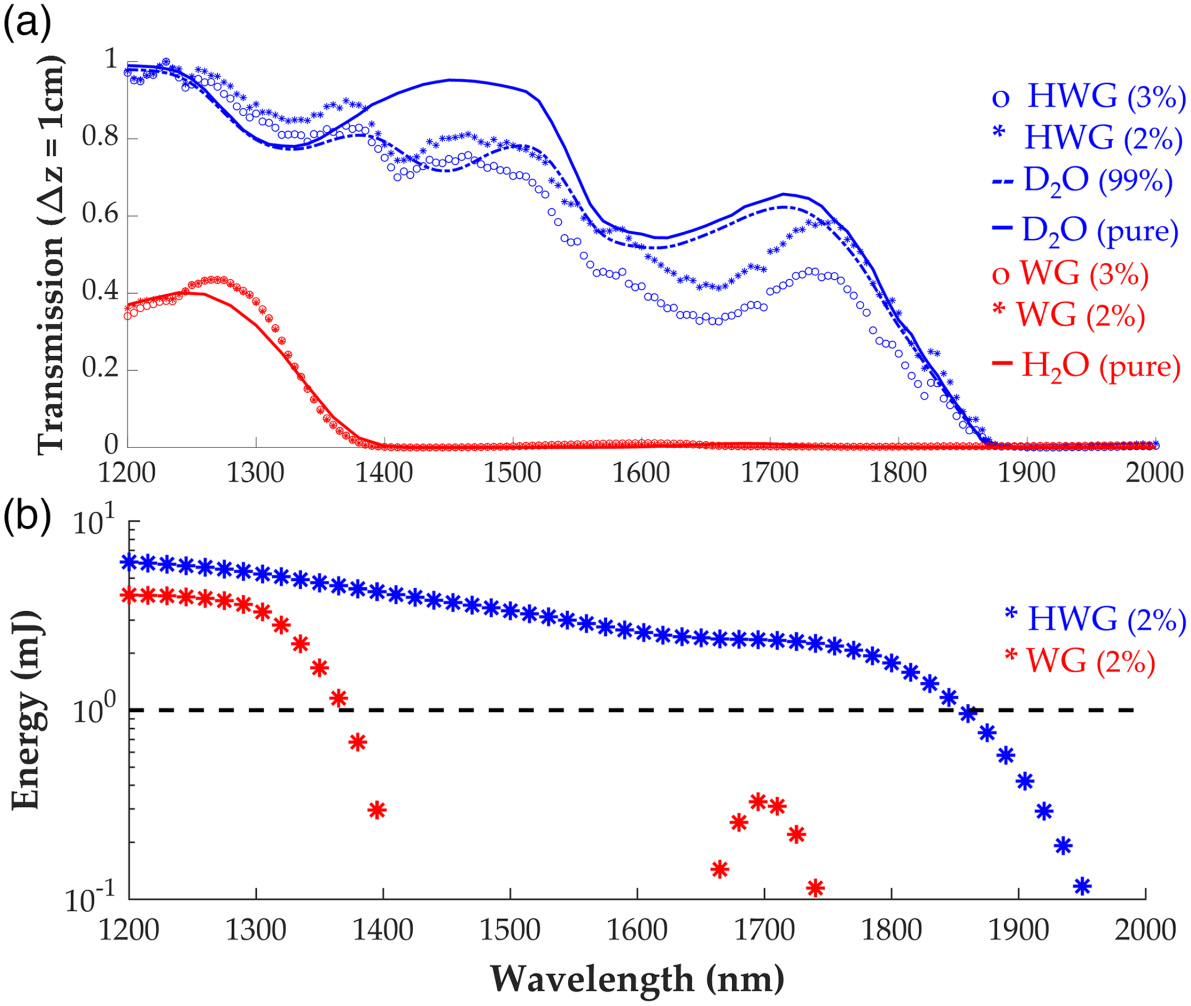
(a) Coupling media PA transmission results. Transmission of HWG and WG at 2% and 3% w/w gellan concentrations are displayed, along with $D_{2}O$ (100% concentration, pure), $D_{2}O$ (99% concentration, 1% $H_{2}O$) and $H_{2}O$ (100% concentration, pure) calculated transmission via absorption coefficients^20^ through an identical pathlength Δz=10  mm. (b) Laser energy measurements of the reflection-mode setup through samples of HWG and WG (thickness ∼5.0  mm) with the horizontal line denoting the cut-off energy threshold of 1 mJ.

Transmission mode data is tabulated and graphed in Fig. 4. The relative PA signals across the band are normalized to black tape for comparing each coupling medium. It is observed that the PA transmission spectrum of HWG (2% and 3% w/w) is similar to the Beer–Lambert signal for 99% heavy water with the same optical pathlength.

Energy measurements of the LAZR-X fiber bundle through 2% w/w HWG and WG are displayed in Fig. 4. We set a criterion that laser illumination reaching the samples with <1  mJ energy was insufficient for producing PA images with adequate SNR. The WG coupling agent, for example, strongly absorbed light above 1350 nm. Light delivery through HWG, on the other hand, maintained sufficient light delivery to the sample (>1  mJ) across the entire SWIR region up to 1850 nm. PA signals were too weak or undetectable outside these cutoff wavelengths. An assessment of the stability of HWG transmission between two time points was also conducted. The HWG sample used in Fig. 4 was tested 60 days after storage in an airtight zip bag stored in a refrigerator at 4°C. Energy measurements after 60 days yielded no significant transmission change (average difference: 1.06±0.49  mJ), demonstrating the stability of HWG with proper storage.

**Fig. 5:**
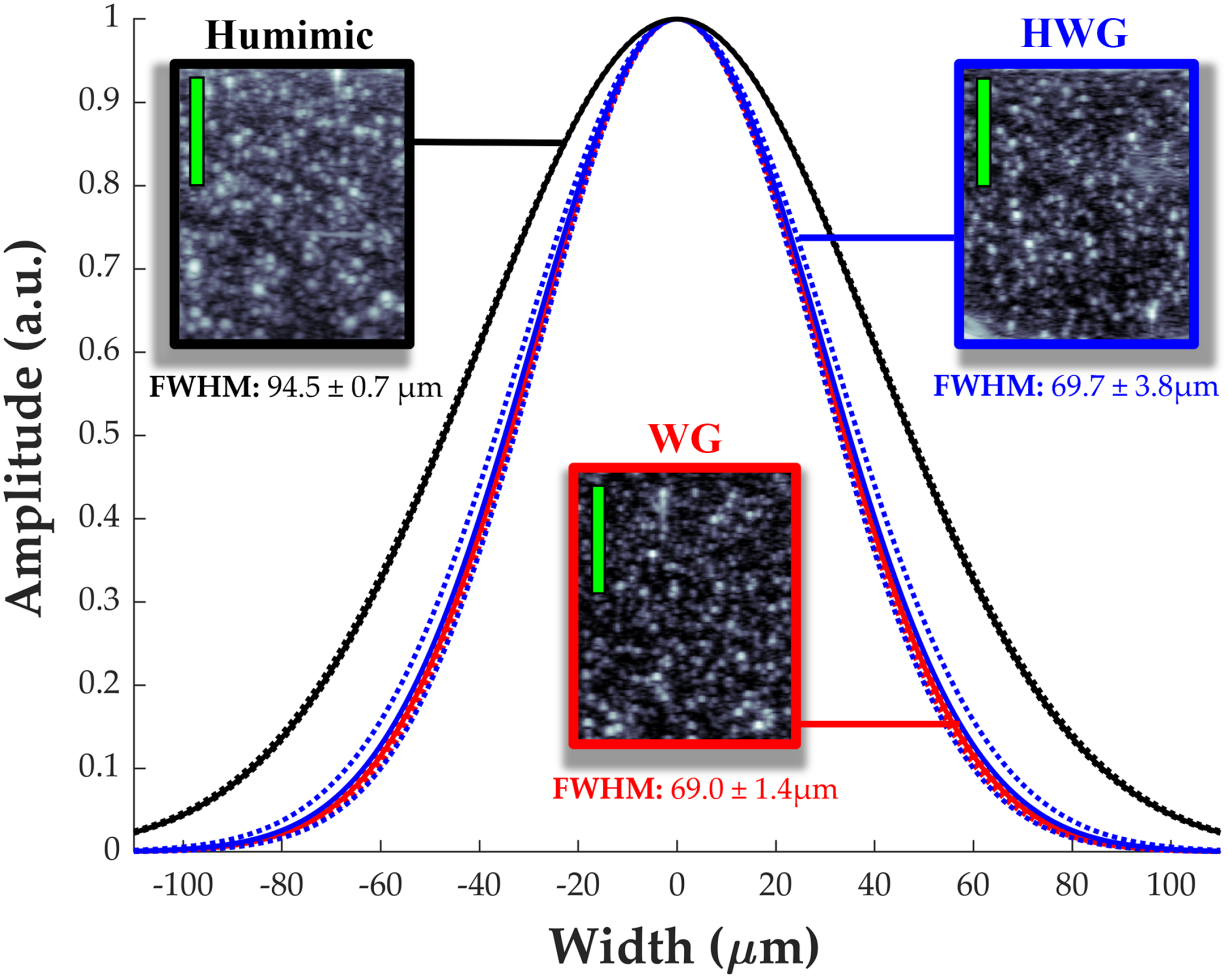
Cross sectional PE images at 25 MHz through graphite phantom in different coupling media. The embedded fine graphite particles served as point scatterers for evaluating the acoustic propagation though the different coupling media: humimic medical rubber (black), HWG (blue), and WG (red). The curves represent the axial PSFs with the FWHMs representing the axial resolutions. Green scale bar denotes 1 mm. Starting depth for each B-mode image is 3 mm from the transducer head.

Figure 5 describes the axial spatial resolution for PE images obtained from the sample with fine graphite particles. Analysis of the PSFs reveals similar axial spatial resolution through WG (69.0±1.4  μm) and HWG (69.7±3.8  μm), indicating that HWG preserves the full acoustic bandwidth of the propagating ultrasound waves similar to a water-based coupling agent. This is not the case with humimic rubber, as the acoustic properties are affected by dispersion and the strong attenuation at high ultrasound frequencies,^32^ resulting in a degradation in axial resolution using the 25 MHz linear array.

### 3.2 Lipid/Water Phantom Imaging

**Fig. 6:**
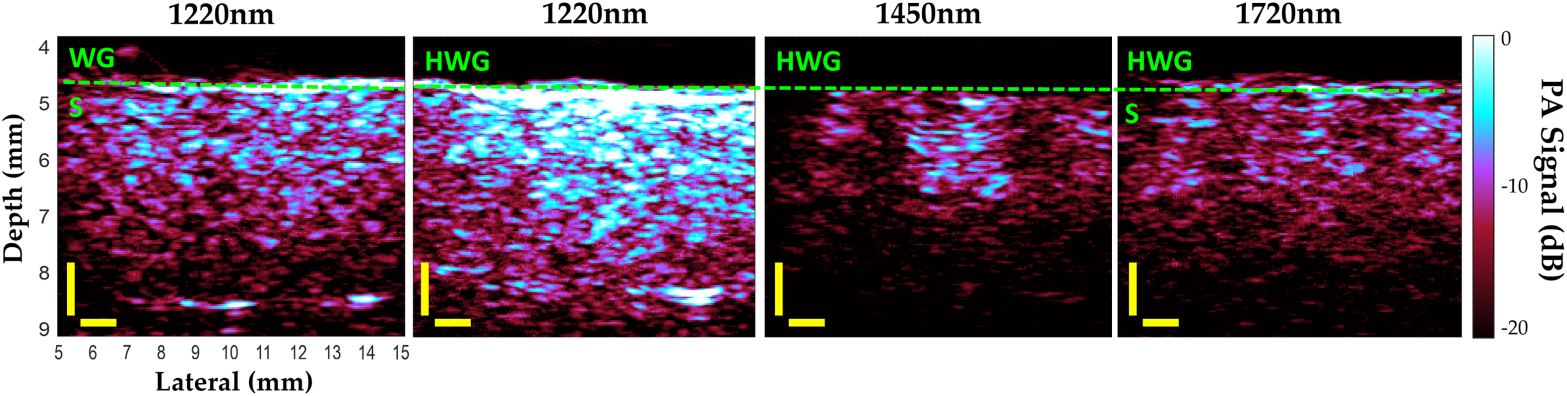
Reflection-mode PA images of lipid/water phantom (S) at peak absorption wavelengths of 1220 nm (lipid), 1450 nm, (water), and 1720 nm (lipid) through 4.5 mm of WG and HWG coupling. Yellow scale bar is 1 mm in both depth and lateral directions. Dashed green line indicates the coupling/sample boundary. No useful PA images were obtained through WG above ∼1350  nm due to the strong absorption of water coupling.

**Fig. 7:**
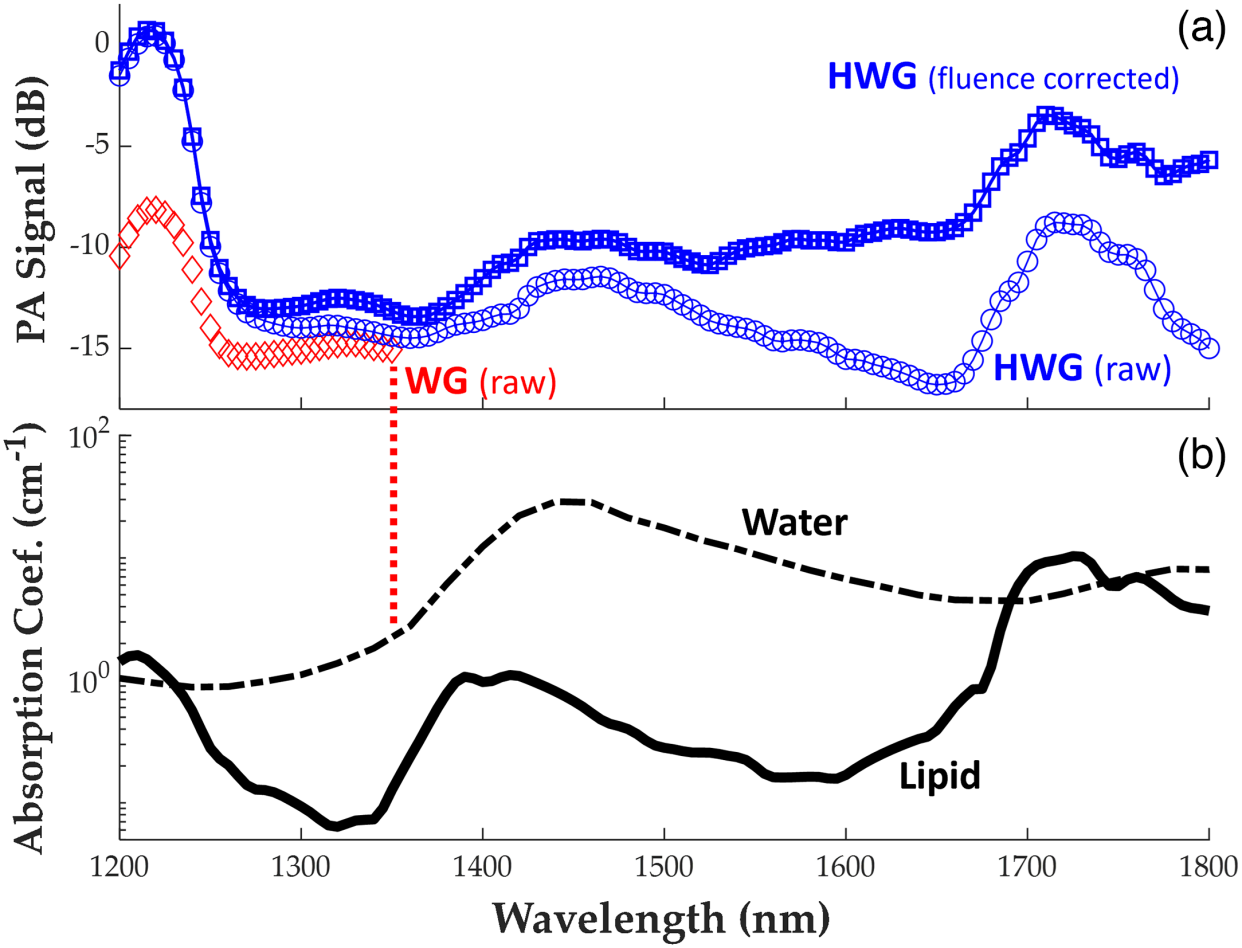
(a) Amplitude of PA signals from the surface of the lipid/water phantom through 5 mm thick HWG and WG coupling; the dark blue plot corrects for wavelength-dependent absorption through HWG (divides raw spectrum by % transmission of HWG at 2% w/w as previously recorded). (b) Published SWIR absorption spectra of lipid and water.^18^^,^^20^ The cut-off energy for detecting PA signals from the sample was defined as 1 mJ for this study (denoted by the red dotted vertical line). Acceptable wavelengths through WG were 1200 to 1350 nm, and 1200 to 1850 nm through HWG.

PA images of the phantoms using the HWG coupling agent were obtained up to 1850 nm as predicted from the absorption coefficients and transmission measurements. On the contrary, water-based gels provided PA images of the sample up to only ∼1350  nm, as depicted in Fig. 6. Signals received past this WG cutoff do not contain any PA information of the sample due to insufficient light reaching the surface. PA signal above the noise floor can be seen using HWG at 1720 nm up to depths of ∼5.0  mm with most of the contrast at this wavelength generated from lipids. PA surface spectra are plotted for the samples in Fig. 7, demonstrating signals above noise for HWG across this full range, including distinct peaks of lipid/water.

At 1220 nm, a 7.5 dB signal increase is observed at the surface of the phantom using HWG compared to WG. This agrees closely with what is expected via the absorption plots in Fig. 4 through a similar optical pathlength at 1220 nm. At a depth of 4.5 mm into the sample, a 4.6 dB signal increase is observed, indicating at depth an SNR increase of nearly 2× is apparent with HWG coupling compared to WG coupling at shorter SWIR wavelengths. For this sample, the noise floor is reached through HWG at a depth of ∼5  mm. At 1720 nm, a 24 dB increase in maximum surface signal is observed when imaged through HWG and fluence corrected as opposed to WG (at the noise floor at this wavelength due to insufficient light delivery to the sample), illustrating the extended usable wavelength range for HWG-enabled PA systems compared to standard coupling methods.

**Fig. 8:**
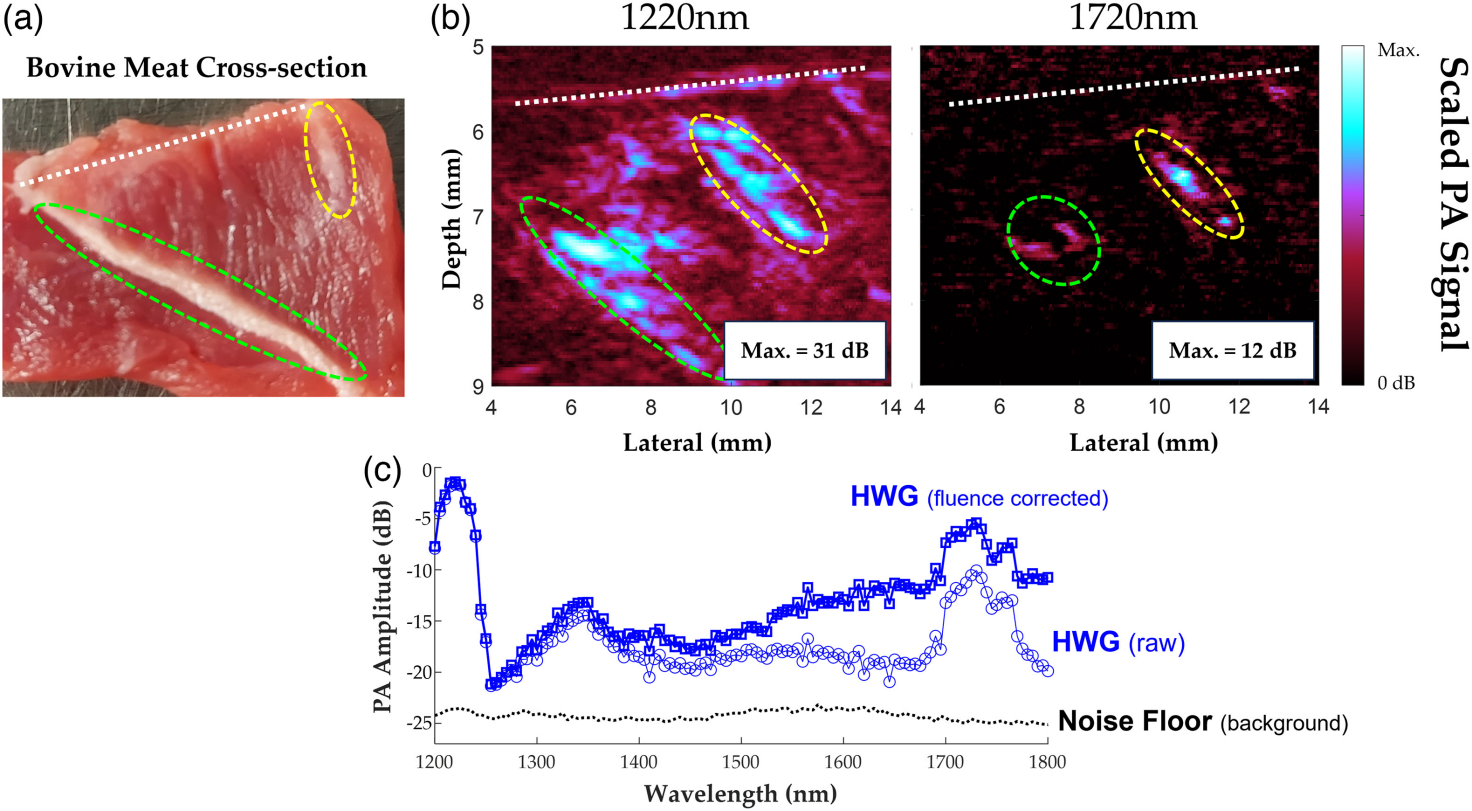
(a) Photograph of bovine muscle sample cross section displaying the approximate slice presented in the PA images. (b) Reflection-mode PA images at absorption peaks of lipid (1220 and 1720 nm) with HWG coupling. Green and yellow regions indicate corresponding pockets of intramuscular fat between the photograph and PA images. The dotted white line indicates the surface of the sample and boundary with coupling medium. Each image is scaled to its own maximum value above the 0 dB noise floor. (c) Average PA spectrum of encircled regions of intramuscular fat using HWG as coupling medium with added fluence correction and noise floor indicated by black dotted line.

Figure 8 displays the PA imaging cross-section of the bovine tissue sample along with spectral data. Even at 1220 nm (obtainable with WG), the ratio of peak PA signal in the green and yellow regions of interest between the HWG and WG images is 3.04× and 1.41×, respectively, indicating a broad increase in SNR when using HWG coupling as opposed to WG.

## 4 Discussion

HWG enables PAI of tissue samples across a broad spectral range in the SWIR (1200 to 1850 nm), whereas WG is limited to wavelengths <1350  nm with poor SNR (Fig. 4). It was anticipated that the predicted transmission spectrum of HWG would be similar to that of heavy water in liquid form at the equivalent concentration (99% pure). The results align with this prediction, as illustrated in Fig. 4. Slight shifts in the transmission peaks in the WG sample compared to baseline are likely due to the bonding mechanism of the low-acyl gellan gum, which has been reported previously.^41^^,^^42^ This effect is also observed with HWG, indicating the bonding effects of gellan and heavy water are similar to that of WG. Akin to water-based coupling, HWG has minimal loss of high frequency acoustic waves, preserving spatial resolution for PE imaging, as shown in Fig. 5. HWG enables the study of tissue constituents in reflection-mode PA setups without sacrificing spatial resolution (Fig. 5). Oil-based rubbers like humimic gels have much stronger acoustic attenuation at high ultrasound frequencies (>20  MHz) due to dispersion and absorption.^32^ In the context of Fig. 5, this implies the PSF produced for humimic gel should be wider in spatial width, which is corroborated with our results using the 25 MHz linear array. The near identical results for HWG and WG signify no loss of PE axial resolution (∼70  μm) through the coupling medium, which is necessary for HWG to be applied to studies with simultaneous high-specificity and high-resolution mapping of constituents, including dual modality PE and PA imaging and spectroscopy in the SWIR. Finally, we demonstrated reflection-mode PAI in the SWIR using the HWG as a solid coupling medium designed for *in vivo* imaging on a commercial scanning system. The HWG interface provides a considerable boost in SNR with penetration into the samples of ∼5.0  mm at 1720 nm. Lipid pool regions in *ex vivo* bovine tissue exhibit a >12  dB increase in the PA signal at 1220 and 1720 nm. This further demonstrates the potential impact of the HWG interface for *in vivo* reflection-mode ultrasound PA imaging studies for detecting and tracking biomarkers like LWC, which have strong and unique optical absorption signatures in the SWIR.

The high-resolution, high-specificity study of tissue constituents such as LWC can henceforth be aided by the addition of HWG to standard reflection-mode PA systems. Current and past systems^27^^,^^28^^,^^43^^,^^44^ used to study constituents may find that the implementation of HWG will result in improved SNR, along with allowing for a larger usable optical bandwidth for spectral characterization and/or unmixing. Other designs that would benefit from improved opto-acoustic coupling include fiber bundle arrangements and optoacoustic in-line reflectors that generally require coupling media. A limitation of this study was that no direct validation with histology was performed to quantify the presence of LWC. Studies which implement spectral-unmixing algorithms^24^^,^^45^^–^^48^ to identify LWC and other constituents in tissue may find a benefit in the increased SNR at chosen wavelengths provided through HWG; however, future validation studies are needed to determine the accuracy of these HWG-enabled spectral unmixing algorithms in the SWIR. One potential drawback of heavy water coupling is its relatively high cost compared to regular water that primarily depends on purity and volume. For this study, we used heavy water with 99% purity with a typical cost of 100 ml ranging from $100 to 300 USD^49^ with some cost fluctuation depending on market value.^50^

Furthermore, clutter effects of PA-induced US waves launched from the sample surface were not explored. This phenomenon is well documented^51^^,^^52^ and known to add discrepancies in PA images, which are not representative of standard PA contrast mechanisms. To further characterize HWG’s potential as a reflection-mode coupling medium, future research should delve into the effects of PA-induced US waves on tissue as a function of coupling absorption. Future work will also include the 3D capability of commercial systems like the Vevo LAZR-X to obtain 3D PA and PE volume data with the HWG retaining contact with the tissue sample or animal. Reflection-mode PA systems with increased capabilities also have promise in clinical environments to aid in the diagnosis of superficial skin lesions relating to skin cancer or tracking the progression of wound healing.^7^^,^^53^^,^^54^ Efforts are underway to incorporate HWG into a novel closed-loop reflection-mode PA system tailored for imaging skin lesions and monitoring wound healing. A patent application has been issued for the HWG interface mechanism as described in this article.^55^ Along with obvious general applications towards PAI, HWG may be used in ultrasound modulation therapy studies; HWG implemented within a proper system design could enable real-time and efficient optical imaging for changes in function during ultrasound modulation therapy.

## 5 Conclusions

In this study, HWG was manufactured and tested as a medium to enable reflection-mode PAI in the SWIR and compared with more common coupling of water-based gelatin and humimic rubber. The HWG interface demonstrated major advantages of optical transmission in the SWIR without sacrificing spatial resolution. We demonstrated reflection-mode PAI for mapping intramuscular lipids in bovine tissue samples at depths of at least 5 mm, which would not be feasible with standard coupling. We envision the interface for reflection-mode PAI in the SWIR to enable *in vivo* studies to quantify and track biomarkers like LWC that are known to change during wound healing and progression of chronic diseases, such as Alzheimer’s disease and skin cancer, as well as their response to therapy.

## Data Availability

All data in support of the findings of this paper are available within the paper.
